# Ataxin-1 regulates epithelial–mesenchymal transition of cervical cancer cells

**DOI:** 10.18632/oncotarget.15319

**Published:** 2017-02-14

**Authors:** A-Ram Kang, Hyoung-Tae An, Jesang Ko, Seongman Kang

**Affiliations:** ^1^ Division of Life Sciences, College of Life Sciences and Biotechnology, Korea University, Seoul 02841, Korea

**Keywords:** ATXN1, cervical cancer, epithelial–mesenchymal transition, notch intracellular domain

## Abstract

The mutant form of the protein ataxin-1 (ATXN1) causes the neurodegenerative disease spinocerebellar ataxia type-1. Recently, ATXN1 was reported to enhance E-cadherin expression in the breast cancer cell line MCF-7, suggesting a potential association between ATXN1 and cancer development. In the present study, we discovered a novel mechanism through which ATXN1 regulates the epithelial–mesenchymal transition (EMT) of cancer cells. Hypoxia-induced upregulation of the Notch intracellular domain expression decreased ATXN1 expression via MDM2-associated ubiquitination and degradation. In cervical cancer cells, ATXN1 knockdown induced EMT by directly regulating Snail expression, leading to matrix metalloproteinase activation and the promotion of cell migration and invasion. These findings provide insights into a novel mechanism of tumorigenesis and will facilitate the development of new and more effective therapies for cancer.

## INTRODUCTION

The 98-kDa, 816 amino acid-long soluble protein ataxin-1 (ATXN1) is found predominantly in the nuclei of neurons and may function as a transcriptional regulator [1]. Notably, the mutant form of ATXN1, which contains an expanded polyglutamine tract, causes spinocerebellar ataxia type-1 (SCA1). Several structural domains of ATXN1 have been identified, including the self-association region and AXH domain, which facilitate protein–protein interactions, and an RNA-binding motif [2, 3]. The AXH domain of ATXN1, which is evolutionarily conserved, may be essential for the pathogenesis of SCA1 in conjunction with the expanded polyglutamine tract [4, 5].

ATXN1 is a component of the Notch signaling pathway [6]. Notch signaling is initiated when the Notch ligand binds to the Notch receptor, which is expressed on neighboring cells. Upon activation, Notch is cleaved via a cascade of proteolytic cleavage steps by the metalloprotease tumor necrosis factor-α-converting enzyme and γ-secretase, thus releasing the Notch intracellular domain (NICD). NICD subsequently translocates into the nucleus and interacts with the transcriptional activator CSL (CBF1/RBPJk in vertebrates, Suppressor of Hairless in Drosophila, Lag-1 in *Caenorhabditis elegans*) to convert the latter into a potent transcriptional activator of Snail [7–10].

Notch signaling reportedly mediates tumor cell migration and invasion [10] induced by low oxygen supply (hypoxia) [10], a critical characteristic of solid tumors [11, 12]. Hypoxia is a powerful and independent prognosticator of poor clinical outcomes in patients with cervical and other types of cancer; in particular, hypoxia can enhance tumor invasiveness, metastasis, and chemotherapeutic resistance [13, 14]. The initial steps of the metastatic process are thought to involve the epithelial–mesenchymal transition (EMT), a highly conserved process by which polarized epithelial cells are converted into mesenchymal cells characterized by the loss of E-cadherin-mediated cell–cell contacts, as well as the acquisition of increased migratory and invasive potential [15–19]. The transcription factors Snail, Slug, Twist, and Zeb1 are negative regulators of E-cadherin expression and are therefore considered potent oncogenic inducers of EMT [20–22].

In our previous study, we reported that the overexpression of ATXN1 enhanced E-cadherin expression at the protein and mRNA levels in MCF-7 breast cancer cells [23]. A potential link between ATXN1 expression (both upregulation and downregulation) and cancer development in humans has been suggested; however, it remains unknown whether ATXN1 acts as a tumor suppressor or an oncogene. In the present study, we aimed to investigate the functional significance of ATXN1 in cervical cancer via studies of its role in the EMT and tumor development. Our results show that ATXN1 plays an important role in cervical cancer cell EMT.

## RESULTS

### Hypoxia-induced upregulation of NICD expression decreases ATXN1 expression

**Figure 1:**
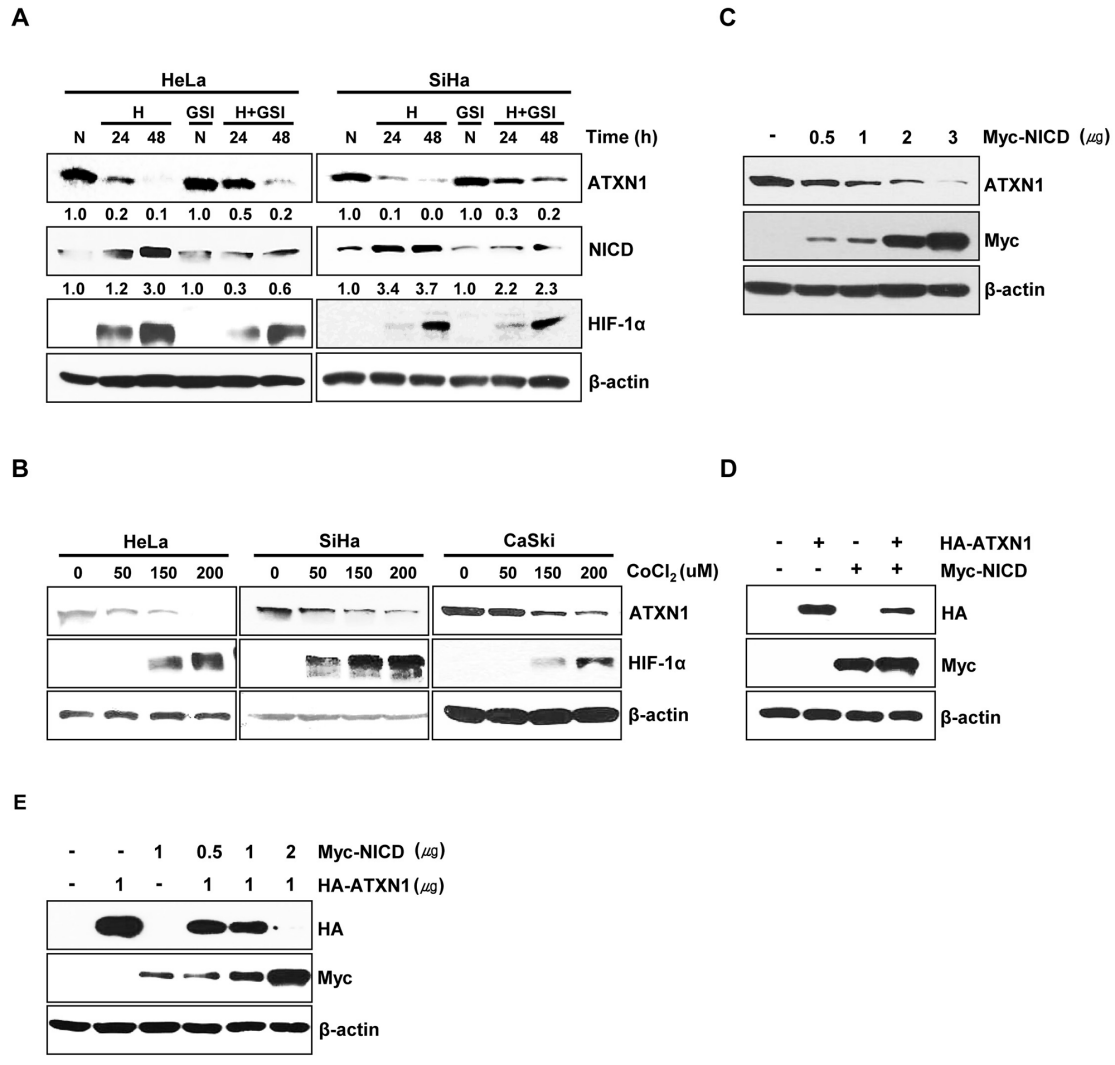
NICD downregulates ATXN1 expression **A**. HeLa and SiHa cells cultured under normoxic or hypoxic conditions for 24 and 48 h in the absence or presence of the γ-secretase inhibitor GSI-DAPT were subjected to a western blotting analysis of ATXN1 expression. Densitometry results of ATXN1 are shown below each lane. ATXN1 expression was normalized to β-actin levels. Numbers indicate the intensity ratio relative to each control lane (1.0) **B**. HeLa, SiHa, and CaSki cells were treated with the indicated concentrations (μM) of CoCl_2_ for 24 h, and lysates were analyzed via western blotting with the indicated antibodies. **C**. HeLa cells transfected with Myc-NICD were subjected to western blotting analysis. **D**. Western blotting analysis of HEK293 cells co-transfected with HA-ATXN1 and Myc-NICD. **E**. Western blotting analysis of HEK293 cells co-transfected with HA-ATXN1 and the indicated amounts of Myc-NICD DNA. H (Hypoxia), N (Normoxia), GSI (γ-secretase inhibitor).

Notch signaling is augmented by hypoxia in various tumor cell lines [10, 24]. We investigated the effect of hypoxia on ATXN1 expression. First, we examined ATXN1 expression in HeLa and SiHa cervical cancer cell line cells cultured under normoxic (21% O_2_) or hypoxic (1% O_2_) conditions. Hypoxia, which was confirmed via expression of the hypoxic marker HIF-1α [25, 26], led to the downregulation of endogenous ATXN1 expression after 24 and 48 h (Figure 1A). Furthermore, our results demonstrated elevated NICD levels in hypoxia and a partial rescue from a decrease in endogenous ATXN1 expression by DAPT GSI, which inhibits the final proteolytic cleavage of the Notch receptor by γ-secretase [27, 28]. We think that DAPT GSI should be not strong enough to completely inhibit the γ-secretase proteins, since NICD was still produced and ATXN1 levels still decreased upon hypoxia in the presence of DAPT.

Further, when we treated HeLa, SiHa, and CaSki cells with CoCl_2_ for 24 h to induce hypoxic conditions, ATXN1 expression decreased greatly (Figure 1B). Notch signaling is activated by hypoxia during tumor progression, leading to proteolytic Notch cleavage and NICD generation [10]. To investigate the relationship between NICD and ATXN1, we transfected HeLa cells with a Myc-NICD construct and found that the expression of endogenous ATXN1 significantly decreased in a dose-dependent manner in the presence of NICD (Figure 1C). Moreover, we performed additional experiments in which HEK293 cells were co-transfected with HA-ATXN1, Myc-NICD, or both and found that exogenous ATXN1 levels also significantly decreased in the presence of NICD (Figure 1D); furthermore, exogenous ATXN1 expression was decreased in the presence of NICD in a dose-dependent manner (Figure 1E). These results suggest that the upregulation of NICD levels in hypoxic cells might suppress ATXN1 expression.

### NICD and MDM2 ubiquitinate and degrade ATXN1

**Figure 2:**
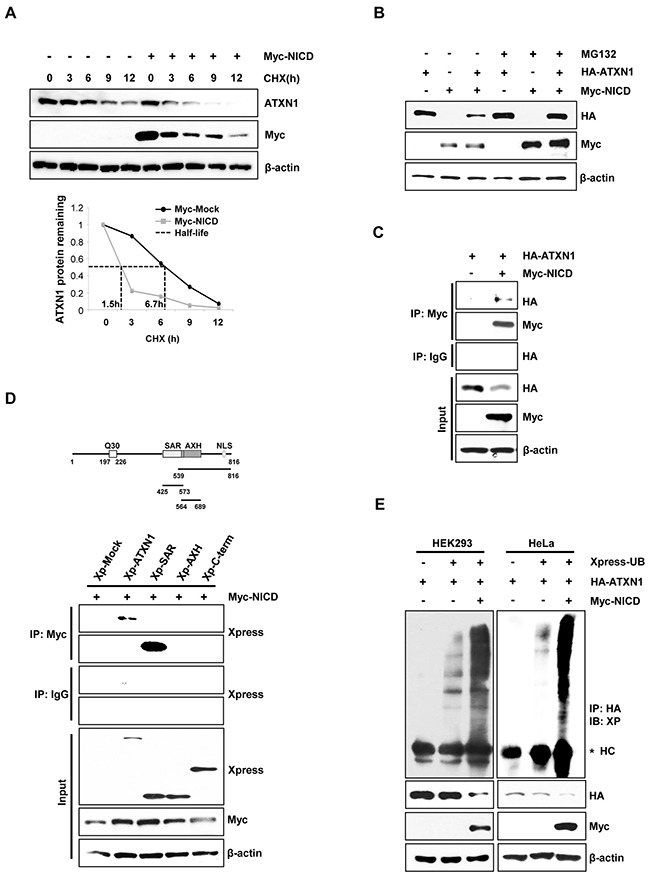
NICD induce ubiquitination and degradation of ATXN1 **A**. Upper panel: Western blotting analysis of HeLa cells transfected with Myc-NICD and treated with 100 μg/ml cycloheximide for various times. Lower panel: Density of ATXN1 was measured and plotted. The half-life of ATXN1 is indicated by dot lines. **B**. Western blotting analysis of HEK293 cells transfected with HA-ATXN1, Myc-NICD, or both, followed by treatment with MG132 for 6 h. **C**. Lysates prepared from HEK293 cells co-transfected with HA-ATXN1 and Myc-NICD were immunoprecipitated using an anti-Myc antibody, and the resulting immunoprecipitates were subjected to western blotting analysis. **D**. Upper panel: ATXN1 deletion constructs. Lower panel: HEK293 cells were co-transfected with Xpress-ATXN1 deletion mutants and Myc-NICD. After transfection, cell lysates were immunoprecipitated with an anti-Myc antibody and subjected to western blotting analysis. **E**. HEK293 and HeLa cells were co-transfected with Myc-NICD and HA-ATXN1 with or without Xpress-Ub and treated with MG132 for 6 h. ATXN1 was immunoprecipitated using an anti-HA antibody. Cell lysates were analyzed via western blotting with the indicated antibodies. IP (Immunoprecipitation), IB (Immunoblot), XP (Xpress), HC (Heavy chain).

To identify the role of NICD in the downregulation of ATXN1, we transfected HeLa cells with Myc-NICD expression vectors. Treatment of these transfectants with cycloheximide (CHX) revealed that the half-life of ATXN1 was shortened in the presence of NICD (Figure 2A). We previously reported that ATXN1 is degraded via the proteasomal degradation pathway [29]. Consistent with this finding, we found that the NICD-induced decrease of ATXN1 levels was inhibited relative to that in control HEK293 cell co-transfected with NICD and ATXN1 constructs treated with the proteasome inhibitor MG132. These findings indicate that NICD may promote the degradation of ATXN1 (Figure 2B).

Immunoprecipitation and western blotting analyses revealed that ATXN1 interacted with NICD in HEK293 cells (Figure 2C). Because ATXN1 contains a self-association region (SAR), ATXN1/HBP1 domain, and C-terminal region [3, 4, 30], we performed immuno-precipitation experiments to determine whether ATXN1 deletion mutants would bind to NICD. Accordingly, we found that the SAR domain bound to NICD (Figure 2D).

ATXN1 is tagged by ubiquitin for proteolytic degradation. Therefore, we co-transfected HEK293 and HeLa cells with HA-ATXN1 and Myc-NICD and detected ubiquitinated HA-ATXN1 in the co-transfectants (Figure 2E). These data support a role of NICD in regulating ubiquitin-dependent ATXN1 degradation.

**Figure 3:**
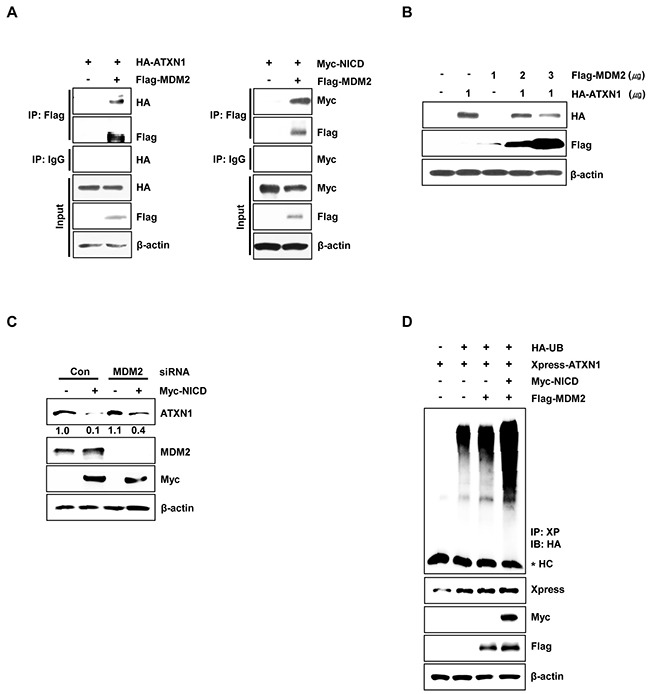
MDM2 promotes ubiquitination and degradation of ATXN1 **A**. Left panel: HEK293 cells were co-transfected with HA-ATXN1 and Flag-MDM2. After transfection, cell lysates were immunoprecipitated with an anti-Flag antibody and subjected to western blotting. Right panel: HEK293 cells co-transfected with Myc-NICD and Flag-MDM2 were lysed, and the lysates were immunoprecipitated with an anti-Flag antibody and subjected to western blotting analysis. **B**. Western blotting analysis of HEK293 cells co-transfected with HA-ATXN1 and Flag-MDM2. **C**. Western blotting analysis of SiHa cells transfected with Myc-NICD in the presence or absence of MDM2 siRNA to determine the expression of Myc-NICD and MDM2. ATXN1 expression was normalized to β-actin levels. Numbers indicate the intensity ratio relative to the control lane (1.0). **D**. HEK293 cells were co-transfected with Myc-NICD, Flag-MDM2, and Xpress-ATXN1, with or without HA-Ub, and treated with MG132 for 6 h. ATXN1 was immunoprecipitated using an anti-Xpress antibody. Cell lysates were subjected to western blotting analysis with the indicated antibodies. HC represents the IgG heavy chain. IP (Immunoprecipitation), IB (Immunoblot), Xp (Xpress), HC (Heavy chain), Con (Control).

We next investigated whether ATXN1 and MDM2 interact. Therefore, we performed immunoprecipitation experiments in HEK293 cells and found that ATXN1 interacts with MDM2; furthermore, NICD also binds to MDM2 (Figure 3A). These results are consistent with a previous study in which NICD was found to bind to MDM2 [31]. Subsequent overexpression of HA-ATXN1 and Flag-MDM2 in HEK293 cells led to dramatic decreases in ATXN1 in the presence of MDM2 in a dose-dependent manner (Figure 3B). These data indicate that NICD induces the degradation of ATXN1 via MDM2-mediated ubiquitination.

Moreover, in SiHa cells co-transfected with Myc-NICD and MDM2 siRNA, NICD-induced ATXN1 degradation was reduced relative to control siRNA-transfected cells (Figure 3C). To determine whether NICD and MDM2 induced ATXN1 ubiquitylation, we co-transfected HEK293 cells with Xpress-ATXN1, Myc-NICD, and Flag-MDM2. We found that the overexpression of NICD and MDM2 increased the ubiquitylation of ATXN1 compared with MDM2 overexpression alone (Figure 3D). These results suggest that NICD and MDM2 synergistically reduce ATXN1 expression at the posttranscriptional level.

### ATXN1 knockdown induces EMT through direct regulation of Snail expression

**Figure 4:**
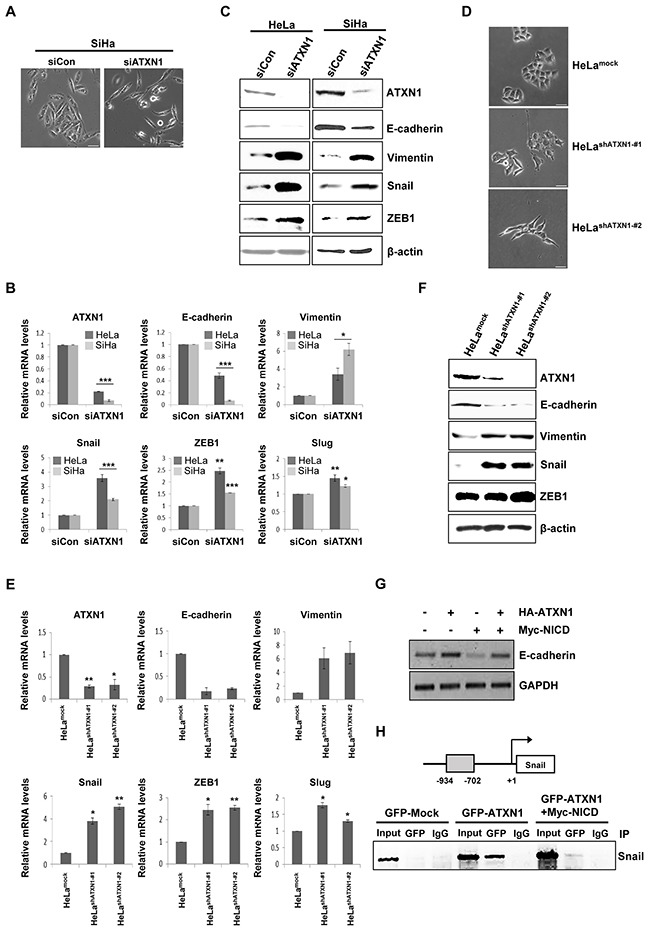
Inhibition of ATXN1 expression induces EMT-like phenotypes **A**. SiHa cells transfected with siATXN1 exhibited morphological changes associated with EMT. **B**. siATXN1 was used to transiently transfect HeLa or SiHa cells, and mRNA levels were estimated using real-time qRT–PCR. **C**. HeLa and SiHa cells were transfected with siCon (sicontrol) or siATXN1, respectively. The levels of EMT markers were determined via western blotting 48 h after transfection. **D**. Light microscopy revealed changes in the morphologies of the HeLa^shATXN1-#1^ and HeLa^shATXN1-#2^ cells. **E**. Real-time qRT–PCR analysis to evaluate the expression levels of EMT marker mRNAs in the stable ATXN1 knockdown cell lines HeLa^shATXN1-#1^ and HeLa^shATXN1-#2^ and control cells. *P<0.05, **P<0.01, ***P<0.001 vs. control group (one-way ANOVA). **F**. Western blotting analysis of EMT markers in HeLa^shATXN1-#1^, HeLa^shATXN1-#2^, and control cells. **G**. RT-PCR analysis of HeLa cells co-transfected with HA-ATXN1 and Myc-NICD. GAPDH was used as a loading control. **H**. Chromatin extracts eluted from SiHa cells expressing GFP-ATXN1 or Myc-NICD were immunoprecipitated with an anti-GFP antibody or normal mouse IgG. Final DNA extractions were amplified using PCR with primers that covered the proximal promoter region of the Snail gene (see Methods). IP (Immunoprecipitation). Each PCR reaction was performed in triplicate. All quantitative data are shown as the means and standard deviations of three independent experiments. *P<0.05, **P<0.01, ***P<0.001, *t* test. Scale bar: 20 μm.

We previously reported that ATXN1 overexpression enhanced E-cadherin expression in the breast cancer cell line MCF-7 [23]. Surprisingly, we found that the SiHa cervical cancer cell line exhibited a mesenchymal-like morphology following ATXN1 depletion (Figure 4A). Downregulation of E-cadherin is one of the critical markers of EMT in human breast cancers [32], and FOXC2, Twist, ZEB1, ZEB2, Slug, and Snail are transcription factors that repress E-cadherin transcription [17, 20, 33]. To investigate whether ATXN1 downregulation would induce EMT in cervical cancer cell lines, we used real-time qRT–PCR to determine the expression levels of EMT markers in HeLa and SiHa cells. Consistent with the observed morphological changes, ATXN1 downregulation in cells transfected with an ATXN1-specific siRNA reduced the expression of mRNA encoding the epithelial marker E-cadherin. In contrast, increased expression of an mRNA encoding the mesenchymal marker vimentin and significantly increased expression of Snail were observed in siATXN1-transfected cells (Figure 4B). The western blot shown in Figure 4C shows that ATXN1 depletion led to the downregulation of E-cadherin and upregulation of vimentin, Snail, and ZEB1.

We next used a lentivirus system to generate HeLa^shATXN1-#1^ and HeLa^shATXN1-#2^ cell lines, which stably expressed ATXN1-specific short hairpin RNAs (shRNAs). A mock lentivirus (empty vector) and lentivirus encoding a nonsilencing shRNA were used as controls. After repeated rounds of puromycin selection, RT–PCR and western blotting analyses revealed stable inhibition of ATXN1 expression in the transduced cell lines HeLa^shATXN1-#1^ and HeLa^shATXN1-#2^ (data not shown). An analysis of the EMT characteristics of these transduced cell lines revealed morphological changes (Figure 4D). As expected, decreased E-cadherin mRNA expression was observed; in contrast, increased expression of vimentin, Snail, Slug, and ZEB1 mRNA was observed (Figure 4E). The increases in Slug and ZEB1 mRNA levels were slight in contrast to the strong increases in Snail mRNA levels. Western blotting analysis of the two transduced cell lines showed that depletion of ATXN1 led to the downregulation of E-cadherin and upregulation of vimentin, Snail, and ZEB1 (Figure 4F). When we co-transfected HeLa cells with HA-ATXN1, Myc-NICD, or both, RT-PCR revealed that the level of E-cadherin mRNA decreased in the presence of NICD, compared with the levels in cells transfected with only ATXN1 (Figure 4G).

Because Snail decreases E-cadherin expression [34, 35], we investigated whether the effects of ATXN1 depletion on Snail expression were regulated at the transcriptional level. Chromatin immunoprecipitation (ChIP) assays of GFP-ATXN1-expressing SiHa cells revealed that ATXN1 was recruited to a region of the Snail promoter (Figure 4H). Further, the recruitment of ATXN1 to the Snail promoter was abrogated in the presence of NICD. Taken together, these results suggest that ATXN1 knockdown induces EMT in cervical cancer cell lines and that the Snail promoter is a direct target of ATXN1.

### Knockdown of ATXN1 induces cell migration and invasion by activating metalloproteinases (MMPs)

**Figure 5:**
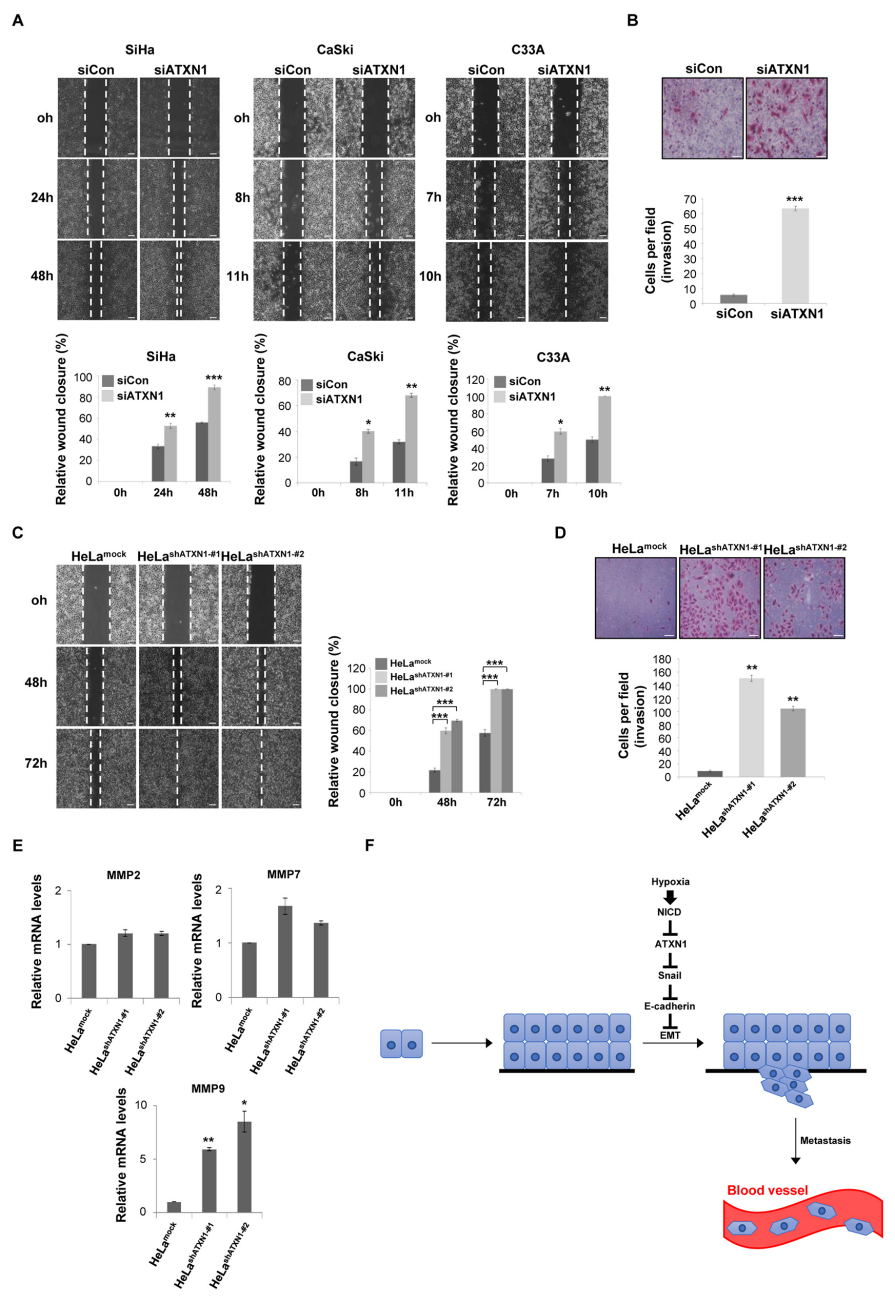
Inhibition of ATXN1 expression increases the migration and invasiveness of cervical cancer cell lines **A**. Upper panel: SiHa, CaSki, and C33A cells were transfected with siCon (sicontrol) or siATXN1 and subjected to a wound-healing assay. Lower panel: Quantification was performed by measuring the migration distances. *P<0.05, **P<0.01, ***P<0.001, *t* test. Scale bar: 100 μm. **B**. SiHa cells were transfected with siCon or siATXN1 and analyzed in a Matrigel invasion assay for 72 h. *P<0.05, **P<0.01, ***P<0.001, *t* test. Scale bar: 20 μm. **C**. The migration of HeLa^shATXN1-#1^, HeLa^shATXN1-#2^, and control cells was assayed in a wound-healing assay. *P<0.05, **P<0.01, ***P<0.001, *t* test. Scale bar: 100 μm. **D**. HeLa^shATXN1-#1^, HeLa^shATXN1-#2^, and control cells were analyzed in a Matrigel invasion assay for 72 h. *P<0.05, **P<0.01, ***P<0.001 vs. control group (one-way ANOVA). Scale bar: 20 μm. **E**. Real-time qRT–PCR analysis of MMP mRNAs in HeLa^shATXN1-#1^, HeLa^shATXN1-#2^, and control cells. All quantitative data are shown as the means and standard deviations of three independent experiments. *P<0.05, **P<0.01, ***P<0.001 vs. control group (one-way ANOVA). **F**. Proposed model for the role of ATXN1 in cervical cancer cell development. Hypoxia-induced upregulation of NICD expression induces reductions in ATXN1 and E-cadherin expression and an increase in Snail expression. Thus, ATXN1 expression decreases in the late stages of tumor development, thus inducing the EMT in cervical cancer cells.

We performed a wound-healing assay to investigate the role of ATXN1 in cell migration. Transfection of the cervical cancer cell lines SiHa, CaSki, and C33A with siATXN1 promoted increased migration relative to controls (Figure 5A). We performed a Matrigel-coated transwell invasion assay to further examine the invasive properties of SiHa cells. Cells transfected with siATXN1 migrated more rapidly and were more invasive than controls (Figure 5B). Furthermore, the migration (Figure 5C) and invasion (Figure 5D) patterns of HeLa^shATXN1-#1^ and HeLa^shATXN1-#2^ cells were similar to those of the siATXN1 transfectants.

Snail induces MMP-9 expression and contributes to cell migration and invasion [36, 37]. MMPs play important roles in tumor growth and the multistep processes of invasion and metastasis, including proteolytic degradation of the extracellular matrix [38–40]. Using real-time qRT–PCR to determine the effects of ATXN1 on MMP expression, we detected increased (>5-fold) levels of MMP-9 in HeLa^shATXN1-#1^ and HeLa^shATXN1-#2^ cells; in contrast, MMP-2 and MMP-7 expression did not change significantly (Figure 5E). Taken together, these results suggest that ATXN1 knockdown facilitates cell migration and invasion by activating MMP-9.

## DISCUSSION

The present study provides new insights into the role of ATXN1 in the pathogenesis of cervical cancer. ATXN1 downregulation contributed to the progression of EMT and induced spindle-like phenotypes. These data indicate that ATXN1 is an important regulator of EMT and cervical cancer progression.

EMT is characterized by loss of cell adhesion, repression of E-cadherin expression, and increased cell mobility. We show here that the depletion of ATXN1 led to the downregulation of E-cadherin expression and upregulation of vimentin, Snail, Slug and ZEB1 expression (Figure 4). In particular, the expression of Snail, a key regulatory inducer of EMT, was significantly increased in HeLa cells engineered to stably express shATXN1. Furthermore, we found that the Snail promoter was a direct transcriptional target of ATXN1, such that stable shATXN1-HeLa transductants exhibited significantly increased migration and invasion compared with controls. These results support the conclusion that ATXN1 depletion upregulates cervical cancer cell EMT.

Moreover, we have demonstrated the ability of NICD to associate with ATXN1 and thus induce the ubiquitination and subsequent proteasomal degradation of ATXN1. The previous finding that NICD does not possess intrinsic E3-ligase activity suggests that NICD may initiate protein degradation or serve as a cofactor. Furthermore, we demonstrated that NICD interacts with MDM2 (Figure 3A) and that MDM2 siRNA-transfected cells exhibited reduced NICD-induced ATXN1 degradation compared with the degradation observed in cells transfected with control siRNA (Figure 3C), suggesting that MDM2 plays a role in NICD-induced ATXN1 degradation.

We accordingly propose the following model for the role of ATXN1 in cervical cancer cell development according to the results presented herein (Figure 5F). Hypoxia, a characteristic feature of locally advanced solid tumors, may increase NICD levels, thus decreasing ATXN1 expression. Accordingly, ATXN1 expression would decrease in the late stages of tumor development. We found that the downregulation of ATXN1 expression induced EMT in cervical cancer cell lines in which the Snail promoter was a direct transcriptional target of ATXN1 and promoted tumor cell migration and invasion. Recently, there have been several reports suggesting that ATXN1 might be involved in tumorigenesis. Zoghbi and her colleagues reported that ATXN1 protein family regulates extracellular matrix remodeling, which imply that the protein family might potentially affect tumorigenesis and cancer metastasis [41]. Analysis on the expression profiles of ATXN1 in various cancer samples using the Oncomine database analysis tool reveals that the level of ATXN1 is significantly downregulated in breast and brain cancer patients. In our previous study, we reported that the ATXN1 regulates E-cadherin expression in MCF-7 breast cancer cells [23].

In summary, we report the discovery of a novel regulatory mechanism that controls ATXN1 expression as well as the mechanism by which ATXN1 regulates cervical cancer cell EMT. Furthermore, we believe that the role of ATXN1 in tumorigenesis is not restricted to cervical epithelial cells. Therefore, regulation of ATXN1 expression may serve as a potential therapeutic target in other cancers.

## MATERIALS AND METHODS

### Cell culture and transfection

The cervical cancer cell lines SiHa, CaSki, and C33A were purchased from the American Type Culture Collection (ATCC, Manassas, VA, USA) and maintained in an RPMI-1640 medium supplemented with 10% fetal bovine serum, streptomycin (100 g/ml), and penicillin (100 U/ml). HeLa and HEK293 cell lines were purchased from ATCC and maintained in a Dulbecco's modified Eagle's medium (DMEM) supplemented with 10% fetal bovine serum, streptomycin (100 g/ml), and penicillin (100 U/ml). Hypoxic conditions were established using an airtight anaerobic incubator (Hypoxia Workstation) to culture cells for 48 h at 37°C in an atmosphere containing 1% O_2_, 5% CO_2_, and 94% N_2_. Transfections were performed in the presence of polyethyleneimine (Sigma-Aldrich, St. Louis, MO, USA) or Lipofectamine (Invitrogen, Carlsbad, CA, USA), as described earlier [29]. Transfection mixtures were combined at a DNA to reagent ratio of 1:3, incubated in serum-free OPTI-MEM (Invitrogen) for 15 min and then applied to the cells.

### Plasmids, reagents, and antibodies

NICD was provided by Dr. Hee-Sae Park (Chonnam National University) [42]. The plasmid Xpress-ATXN1 was generated by subcloning ATXN1 into the *Eco*RI/*Xho*I sites of the pcDNA3.1-HisC-Xpress vector, a modified version of pcDNA3.1. The plasmid HA-ATXN1 was described earlier [43]. The siGENOME SMARTpool contains a mixture of four SMART selection-designed siRNAs (siATXN1) that target the gene encoding ATXN1 (Dharmacon, Lafayette, CO, USA). Lentiviral particles containing a pLKO.1 transfer vector and shRNA specific for ATXN1 mRNA (Thermo Fisher Scientific, Waltham, MA, USA) were prepared using HeLa cells. Gamma-secretase inhibitor IX (DAPT GSI) was purchased from Selleck Chemical (Houston, TX, USA). CoCl_2_ was dissolved in dimethyl sulfoxide (DMSO; Sigma-Aldrich). Primary antibodies against the following proteins were used: ATXN1, HIF-1α, Snail, and ZEB1 (Cell Signaling Technology, Danvers, MA, USA); E-cadherin, NICD, and MDM2 (Abcam, Cambridge, UK); vimentin, GFP, HA, and c-Myc (Santa Cruz Biotechnology, Inc., Dallas, TX, USA); Xpress (Invitrogen); and Flag (Sigma-Aldrich).

### Protein degradation assay

HeLa cells were transiently transfected with the pCS2 vector with or without Myc-NICD. After a 24-h incubation, the cells were chased with 100 mg/ml of CHX for different time periods. Cells were collected at the indicated times and processed for immunoblotting using antibodies against ATXN1, c-Myc, and β-actin.

### Immunoprecipitation and western blotting

Co-immunoprecipitation experiments were performed as described earlier [44]. Briefly, HEK293 cells were transiently co-transfected with HA-ATXN1 or Myc-NICD. After 48 h, cells were harvested and lysed on ice for 60 min in 0.7% NP-40 lysis buffer supplemented with protease inhibitors (10 μg/ml aprotinin, 10 μg/ml leupeptin, and 2 mM PMSF). The resulting lysates were centrifuged at 13,000×*g* for 20 min at 4°C, and the supernatants were incubated with the anti-c-Myc antibody at 4°C overnight. Protein G-Sepharose beads (GE Healthcare, Little Chalfont, UK) were then added, and bead-bound proteins were analyzed using sodium dodecyl sulfate (SDS)–polyacrylamide gel electrophoresis. The proteins were electrophoretically transferred to nitrocellulose membranes (Whatman/GE Healthcare) and probed with the appropriate antibodies. The immune complexes were detected with an enhanced chemiluminescent immunoblotting system (Amersham Pharmacia Biotech/GE Healthcare) according to the manufacturer's instructions [45].

### *In vivo* ubiquitylation assay

HEK293 and HeLa cells were transiently transfected with HA-ATXN1, Xpress-Ub, or Myc-NICD for 24 h, followed by incubation with the proteasome inhibitor MG132 (10 μM) for 6 h. Cells were lysed for 60 min at 4°C in a RIPA buffer (20 mM Tris-Cl, 150 mM NaCl, 0.1% SDS, 1% Triton X-100, 1% sodium deoxycholate, pH 7.5) containing the indicated protease inhibitor. Protein concentrations were determined using the Bio-Rad Protein Assay Kit (Bio-Rad Laboratories, Hercules, CA, USA). Cell lysates were immunoprecipitated using an anti-HA antibody, after which the precipitated proteins were subjected to western blotting and the blots were probed using an anti-Xpress antibody.

### Quantitative real-time PCR

We used TRIzol reagent (Invitrogen) to isolate total RNA from HeLa and SiHa cells transfected with either ATXN1 or control siRNA. qRT–PCR reactions to synthesize cDNA from 1 μg of total RNA were performed using the First-Strand Synthesis System (Invitrogen) and an oligo(dT)_20_ primer. E-cadherin, ATXN1, Snail, Slug, ZEB1, vimentin, MMPs, and GAPDH cDNAs were amplified using the SYBR Green Real-time PCR Master Mix and a LightCycler 480 instrument (Roche, Basel, Switzerland). Forward and reverse primer sequences are available upon request.

### Chromatin immunoprecipitation

ChIP assays were performed as described earlier [23]. Briefly, SiHa cells were transfected for 48 h with 3 μg of GFP-ATXN1, Myc-NICD, or empty vector DNAs; the subsequent crosslinking of cellular DNA was induced using 1% formaldehyde and terminated by adding 0.2 M glycine. Pellets prepared via centrifugation were washed twice with ice-cold Tris-buffered saline and incubated three times with MC lysis buffer (10 mM Tris-Cl [pH 7.5], 10 mM NaCl, 3 mM MgCl_2_, and 0.5% NP-40) to disrupt the cells and generate nuclear pellets; these were resuspended in MNase buffer (10 mM Tris-Cl [pH 7.5], 10 mM NaCl, 3 mM MgCl_2_, 1 mM CaCl_2_, 4% NP-40, and 1 mM PMSF), treated with 2 mM PMSF, 1× protease inhibitors, 1% SDS, and 200 mM NaCl, and mixed well. Sonication was used to shear the resuspended pellet and reduce the DNA fragment size to approximately 500 bp. After removing cellular debris, chromatin samples were diluted (1:4) by adding FA lysis buffer (50 mM HEPES [pH 7.5], 150 mM NaCl, 1 mM EDTA, 1% Triton X-100, 0.1% sodium deoxycholate, and 0.1% SDS) containing 2 mM PMSF and 1× protease inhibitors. Ten percent of the precleared chromatin was used as input, and the remaining supernatant was immunoprecipitated using an anti-GFP antibody for 4 h at 4°C. Immunoprecipitated samples were then incubated with protein G-Sepharose beads (GE Healthcare) for 2 h at 4°C. DNAs and proteins that associated nonspecifically with the protein G-Sepharose beads were removed by washing twice with FA lysis buffer/0.15 M NaCl, once with FA lysis buffer/0.5 M NaCl, ChIP washing buffer (10 mM Tris-Cl [pH 8.0], 0.25 M LiCl, 1 mM EDTA, 0.5% NP-40, and 0.5% sodium deoxycholate), and TE buffer (10 mM Tris-Cl [pH 8.0 and 1 mM EDTA). The beads were then resuspended in ChIP elution buffer (50 mM Tris-Cl [pH 7.5], 10 mM EDTA, and 1% SDS) for 10 min at 65°C. The eluted protein–DNA complexes were incubated for 2 h at 42°C in the presence of 2 mg/ml proteinase K, followed by an overnight incubation at 65°C to reverse the crosslinks. The DNA was extracted with phenol, precipitated from the aqueous phase using ethanol, and PCR amplified using Snail-specific primers to detect the human Snail promoter region, as described earlier [10] (primer sequences: 5′-ATCCCTGGAAGCTGCTCTCT-3′ and 5′-TCTGGTCCAGTGAGGGAG-3′). The PCR cycling conditions were as follows: 95°C for 5 min; 35 cycles at 94°C for 20 s, 56.9°C for 20 s, 72°C for 20 s; and 72°C for 5 min. The amplified DNA was electrophoresed through a 2% agarose gel and visualized using ethidium bromide.

### Cell migration and invasion assays

For wound-healing assays, SiHa, CaSki, and C33A cells were transfected with an ATXN1 siRNA or control siRNA and allowed to adhere to the surfaces of six-well plates; subsequently, 10-μl pipette tips were used to introduce longitudinal straight scratches in the cell layers. The medium and cell debris were aspirated and replaced with a fresh culture medium. After wounding, plates were imaged using a Zeiss inverted microscope equipped with a 5× phase-contrast objective.

The invasiveness of SiHa cells was monitored using BD BioCoat Matrigel Invasion Chambers (BD Biosciences). Cells (5 × 10^4^ in 100 μl of serum-free medium) were seeded into the upper chambers of transwell dishes; the lower chambers were filled with 0.8 ml of RPMI-1640 containing 10% fetal bovine serum. The dishes were then incubated for 72 h at 37°C, after which the membranes were fixed in methanol and stained with hematoxylin and eosin. Cells remaining on the upper surface of the membrane were removed, and invasive cells were counted in four randomly selected high-power fields per membrane using a light microscope. Each sample was assayed in triplicate, and the assays were performed three times.

### Statistical analysis

Differences between the various experimental groups were calculated using the two-tailed independent Student's *t* test or one-way ANOVA. *P*-values <0.05 were considered statistically significant.

National R&D Program for Cancer Control, the Ministry of Health & Welfare, Republic of Korea (1320010)

National Research Foundation of Korea (NRF) grant (2015R1A4A1041919).

## SUPPLEMENTARY MATERIALS FIGURES


