# Supplementary material for: Ataxin-1 regulates epithelial–mesenchymal transition of cervical cancer cells
Source: Oncotarget. 2017 Feb 14;8(11):18248–59. doi: 10.18632/oncotarget.15319 (PMC5392324; doi:10.18632/oncotarget.15319)
Supplement: Supplementary file 1 [file oncotarget-08-18248-s001.pdf]

Ataxin-1 regulates epithelial–mesenchymal transition of cervical cancer cells

SUPPLEMENTARY METHODS

For wound-healing assays, $HeLa^{shATXN1\text{-}\#1}$, $HeLa^{shATXN1\text{-}\#2}$, and control cells were allowed to adhere to the surfaces of six-well plates. After a pretreatment with mitomycin C (10 μg/ml) in DMEM for 2 h, 10-μl pipette tips were used to introduce longitudinal straight scratches in the cell layers. After wounding, plates were imaged using a Zeiss inverted microscope equipped with a 5× phase-contrast objective. Representative wound healing images were taken at the time of the scratch and 0–72 h after the wound scratch in stable HeLa cells.

SUPPLEMENTARY FIGURE

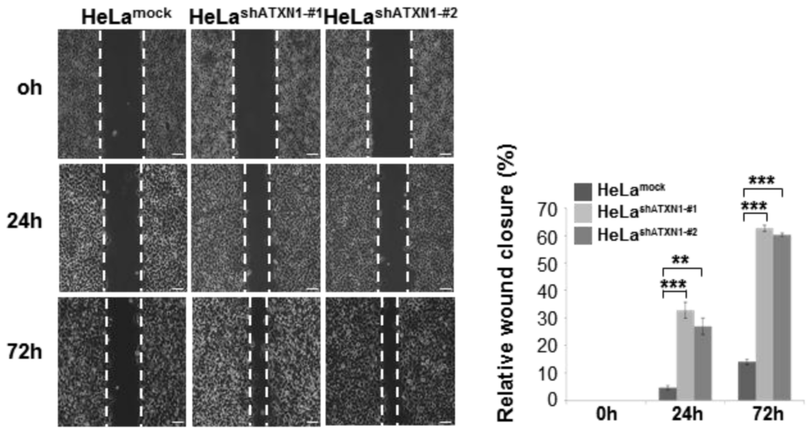

Supplementary Figure 1: The migration of $HeLa^{shATXN1\text{-}\#1}$, $HeLa^{shATXN1\text{-}\#2}$, and control cells was assayed in a wound-healing assay. * $P<0.05$, ** $P<0.01$, *** $P<0.001$, *t* test. Scale bar: 100 μm.
